# BCR::ABL1 tyrosine kinase inhibitors hamper the therapeutic efficacy of blinatumomab in vitro

**DOI:** 10.1007/s00432-022-04039-5

**Published:** 2022-05-13

**Authors:** Joseph Kauer, Melanie Märklin, Martin Pflügler, Sebastian Hörner, Clemens Hinterleitner, Claudia Tandler, Gundram Jung, Helmut R. Salih, Jonas S. Heitmann

**Affiliations:** 1 Departament of Immunology, Interfaculty Institute for Cell Biology, University of Tübingen, Tübingen, Germany; 2 German Cancer Consortium (DKTK) and German Cancer Research Center (DKFZ), Partner Site Tübingen, Tübingen, Germany; 3 Clinical Collaboration Unit Translational Immunology, German Cancer Consortium (DKTK), Department of Internal Medicine, University Hospital Tübingen, Otfried-Müller-Str. 10, 72076 Tübingen, Germany; 4 Department of Medical Oncology and Pneumology (Internal Medicine VIII), University Hospital Tübingen, Tübingen, Germany; 5 DFG Cluster of Excellence 2180 ‘Image-Guided and Functional Instructed Tumor Therapy’ (IFIT), University of Tübingen, Tübingen, Germany

**Keywords:** Acute lymphoblastic leukaemia, BCR::ABL1, Blinatumomab, Tyrosine kinase inhibitors

## Abstract

**Purpose:**

Acute B-lymphoblastic leukemia (B-ALL) is a malignant disease characterized by accumulation of clonal immature lymphocytes in the bone marrow and peripheral blood. The approval of BCR::ABL1 tyrosine kinase inhibitors (TKI) such as imatinib, dasatinib, nilotinib and ponatinib marked a milestone in targeted therapy only for a subset of patients carrying the translocation t(9;22)(q34;q11). Immunotherapy with the bispecific antibody (bsAb) blinatumomab targeting CD19xCD3 revolutionized treatment of all B-ALL cases. The combination of both TKI and bsAb, so-called “dual targeting”, is currently under clinical investigation, although TKI might influence T cell effects.

**Methods:**

We here investigated the combination of different TKI and blinatumomab in BCR::ABL1^+^ and BCR::ABL1^−^ B-ALL cell lines and primary samples regarding T cell proliferation, differentiation, cytokine release and killing of tumor cells.

**Results:**

In vitro analysis revealed profound reduction of T cell proliferation, differentiation, cytokine release and killing of tumor cells upon application of BCR::ABL1 TKI with blinatumomab. Inhibition was more pronounced with dasatinib and ponatinib compared to nilotinib and imatinib. T cell signalling after CD3 stimulation was impaired by TKI mirrored by inhibition of LCK phosphorylation. This known off-target effect might influence the efficacy of bsAb therapy when combined with BCR::ABL1 TKI.

**Conclusion:**

In conclusion, we propose that nilotinib and imatinib might also be suitable substances for combination with blinatumomab and suggest evaluation in clinical trials.

**Supplementary Information:**

The online version contains supplementary material available at 10.1007/s00432-022-04039-5.

## Introduction

Bispecific antibodies (bsAbs) improve treatment of hematological diseases, in particular since blinatumomab entered clinical routine for the treatment of acute B-cell lymphoblastic leukemia (B-ALL), a disease characterized by rapid accumulation of clonal CD19^+^ immature B cells in the bone marrow and peripheral blood (Franquiz and Short 2020). Blinatumomab binds CD19 and CD3 and thereby engages T cells with leukemic blasts, thus inducing a target cell-restricted tumor cell lysis (Bargou et al. 2008). This bsAb was initially approved for refractory and relapsed disease, but in the following years showed promising results in the frontline setting and combinatorial regimens (Gökbuget et al. 2018). However, uncertainty still remains regarding the optimal combination and sequence of blinatumomab, cytotoxic chemotherapy and other emerging agents. Besides chemotherapy consisting of, e.g., cyclophosphamide and anthracyclines, targeted therapy with tyrosine kinase inhibitors (TKI) was approved for a subset of B-ALL cases (Druker et al. 2001). Initially developed for chronic myelogenous leukemia (CML), which carries the translocation t(9;22)(q34;q11) leading to the BCR::ABL1 fusion protein, inhibitory reagents such as dasatinib (DASA), nilotinib (NILO), ponatinib (PONA) and imatinib (IMA) are used for treatment of BCR::ABL1^+^ B-ALL, a subtype comprising approximately 30% of cases (Hunger and Mullighan 2015; Komorowski et al. 2020). The first approved BCR::ABL1 inhibitor, IMA (Druker et al. 2006), was later complemented by next-generation inhibitors to provide treatment options for IMA-resistant disease (Druker et al. 2006; Talpaz et al. 2006). The second and further generation inhibitors display higher potency regarding BCR::ABL1 inhibition and have a different toxicity profile than IMA (Rix et al. 2007; Weisberg et al. 2007; Kantarjian et al. 2010; Saglio et al. 2010; Krusch and Salih 2011). Of note, TKIs may, besides affecting tumor cells, also inhibit signaling events responsible for activation of immune effector cells, thus pointing to a novel challenge which arises with increasing treatment options. One example constitutes the affection of other kinases like the stem cell factor receptor (cKIT), Src kinases or platelet-derived growth factor receptors (PDGFR), which are involved in the activation of immune effector cells (Mannaioni et al. 1997; Hantschel et al. 2008). Most importantly, the majority of aforementioned TKIs have been reported to inhibit T cell activation and proliferation (Appel et al. 2005; Chen et al. 2008; Schade et al. 2008a, b). Nevertheless, the combination of blinatumomab and DASA exhibited tremendous success in refractory and relapsed B-ALL with 95% overall survival 18 months after initiation of therapy (Foa et al. 2020). Trials combining blinatumomab with other TKIs, e.g., PONA (NCT03263572) are ongoing, the ideal BCR::ABL1 inhibitor to be used in combination regimes is not yet identified (Assi et al. 2017; Chiaretti et al. 2019; King et al. 2019).

Here, we evaluated the combination of bsAb with four different TKIs to unravel the in vitro potential of the combination to provide further evidence for a clinical trial. In addition, we identified potential drawbacks of the combinatorial treatment.

## Results

### BCR::ABL1 inhibitors interfere with blinatumomab-induced T cell activation and proliferation

**Fig. 1:**
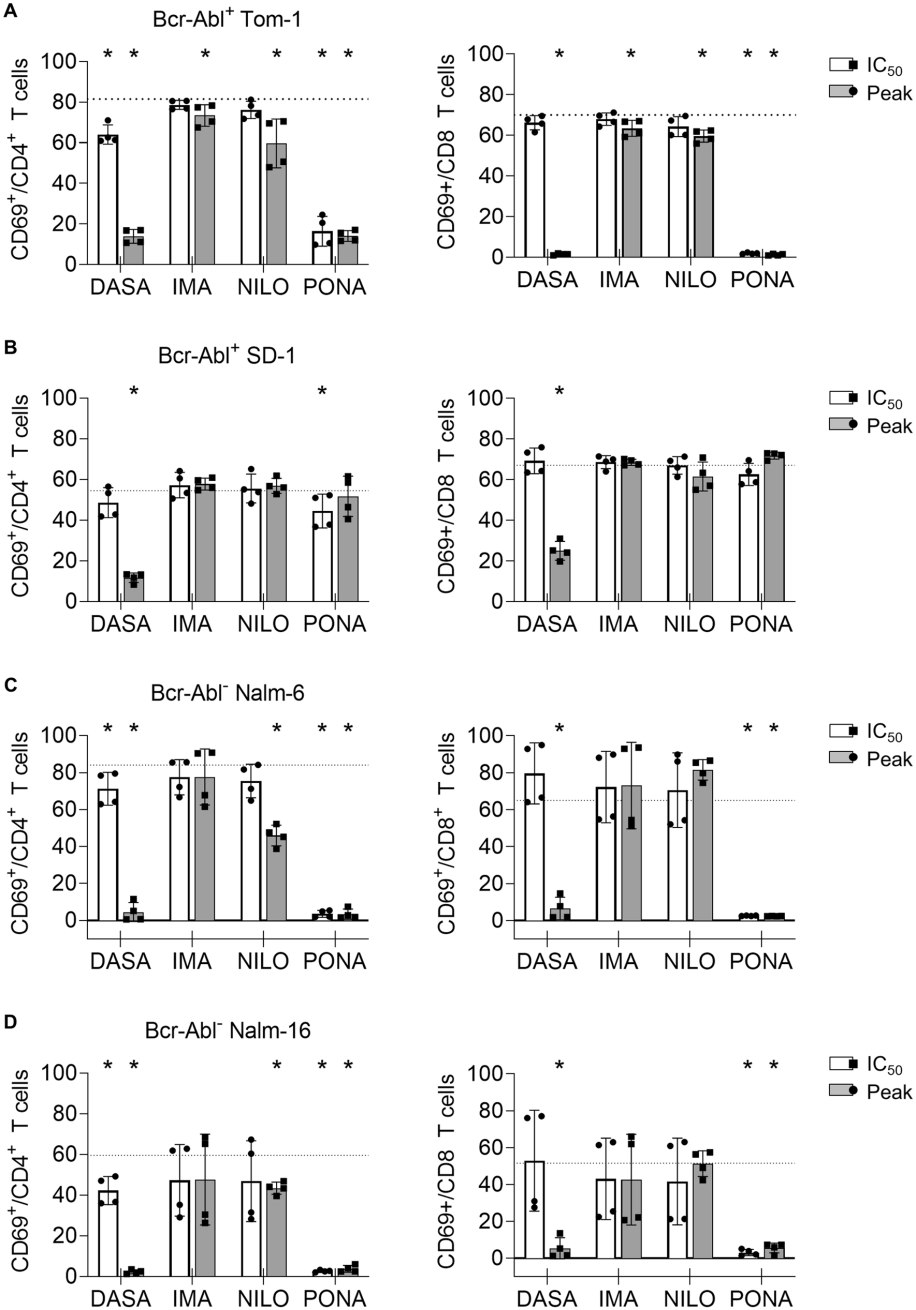
Impact of BCR::ABL1 TKI on T cell activation induced by blinatumomab and B-ALL cells. CD69 expression on CD4^+^ and CD8^+^ T cells was analyzed by flow cytometry in 3 day coculture assays (*n* = 4 donors) with 100,000 PBMC, 100,000 ALL cells and blinatumomab at 1 ng/ml. The following ALL cell lines were utilized: **A** BCR::ABL1^+^ TOM-1, **B** BCR::ABL1^+^ SD-1, **C** BCR::ABL1^−^ Nalm-6, **D** BCR::ABL1^−^ Nalm-16. The dotted lines correspond to the CD69 expression levels with PBMC, ALL cell line and blinatumomab. Statistical analysis with Mann–Whitney *U* test. *p < 0.05

**Fig. 2:**
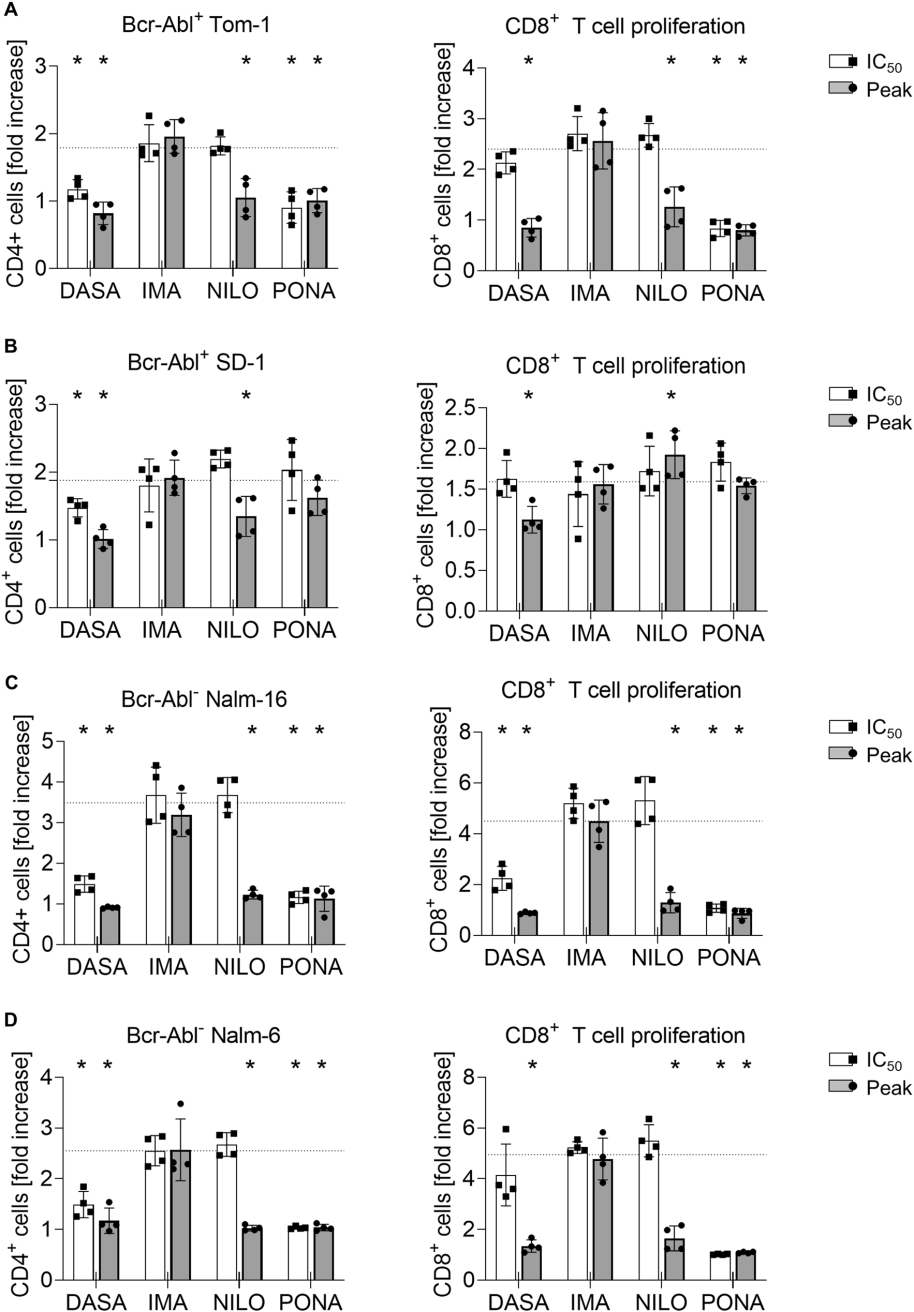
Impact of BCR::ABL1 TKI on T cell proliferation induced by blinatumomab and B-ALL cells. Proliferation of CD4^+^ and CD8 T cells (depicted as fold increase) was analyzed by flow cytometry in 3 day coculture assays (*n* = 4 donors) with 100,000 PBMC, 100,000 ALL cells and blinatumomab at 1 ng/ml. The following ALL cell lines were utilized: **A** BCR::ABL1^+^ TOM-1, **B** BCR::ABL1^+^ SD-1, **C** BCR::ABL1^−^ Nalm-6, **D** BCR::ABL1^−^ Nalm-16. The dotted lines correspond to the proliferation seen with PBMC, ALL cell line and blinatumomab. Statistical analysis with Mann–-Whitney *U* test. *p < 0.05

Both CD19-directed therapies and tyrosine kinase inhibition targeting BCR::ABL1^+^ blasts show great efficacy in B-ALL. Combinational therapy therefore might be an option for high-risk patients. However, the effect of BCR::ABL1 TKI on blinatumomab therapy remained unclear. To address this question, we performed flow cytometry-based lysis assays using four ALL cell lines: BCR::ABL1^+^ Tom-1, BCR::ABL1^+^ SD-1, BCR::ABL1^−^ Nalm-6 and BCR::ABL1^−^ Nalm-16. Exemplary gating strategies are depicted in Supplementary Figure S1A-D. After 3 days of incubation with blinatumomab, approximately 60–80% of CD8^+^ T cells were activated as mirrored by CD69 expression. Potent inhibition of T cell activation was seen upon treatment with DASA at plasma peak levels and with PONA even at IC_50_ levels. IMA and NILO showed only moderate effects and in most cases did not inhibit T cell activation by blinatumomab (Fig. 1). T cell activation in the presence of SD-1 cells might be preserved due to increased unspecific immunogenicity of this cell line. Since T cell expansion is a prerequisite for successful bsAb therapy, the influence of TKI on blinatumomab-mediated T cell proliferation was investigated. Blinatumomab induced a 2 to 5-fold increase in absolute CD4^+^ and CD8^+^ T cell numbers upon three day cocultures with PMBC and ALL cell lines. Upon concomitant treatment with DASA and PONA even at IC_50_ levels, proliferation was significantly inhibited (Fig. 2). NILO hampered T cell proliferation only at plasma peak doses and to a lesser extent, while IMA did not show any negative effect on T cell proliferation. This was confirmed by thymidine incorporation assays (Supplementary Figure S2A).

### BCR::ABL1 inhibitors limit T cell differentiation and cytokine release

**Fig. 3:**
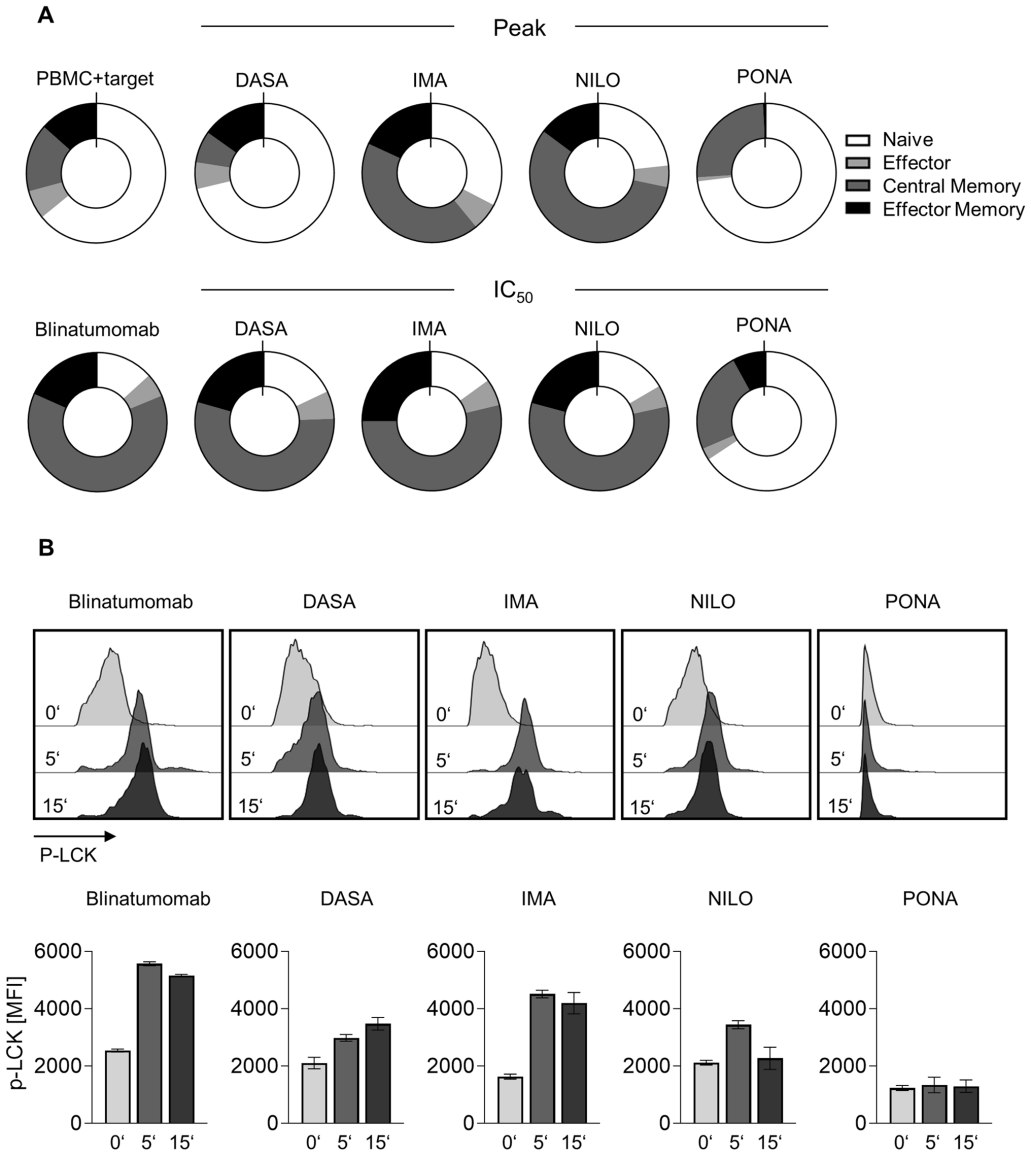
Impact of TKI on blinatumomab-induced differentiation of T cells and T cell signaling**. A** Proportion of different CD8^+^ T cell subsets after 3 day coculture assays (*n* = 4 donors) with 100,000 PBMC, 100,000 BCR::ABL1^+^ SD-1 cells and blinatumomab at 1 ng/ml. **B** Phosphorylation of LCK (p-Y394) on T cells after 0, 5 and 15 min of stimulation with plate-coated blinatumomab was determined by phosphoflow. Pretreatment with TKI was performed overnight prior to bsAb exposure. Exemplary data from one out of three experiments is depicted. *p < 0.05

Since the efficacy of blinatumomab is thought to rely on expansion of effector memory T cells (Klinger et al. 2012), we investigated whether the TKIs interfered with blinatumomab-induced T cell differentiation. By staining for CD45RA, CD45RO and CD62L, T cell subsets were identified after 3d stimulation with blinatumomab in the presence of BCR::ABL1^+^ TOM-1 cells (exemplary gating strategy depicted in Supplementary Figure S1E). Incubation with blinatumomab resulted in T cell differentiation into central memory T cells (CD45RO^+^CD62L^+^), effector memory T cells (CD45RO^+^CD62L^−^), and to a lesser extent effector T cells (CD45RA^+^CD62L^−^). IMA and NILO at peak concentrations had only minor effects on differentiation, as mirrored by a reduction of central memory T cells. DASA led to profoundly reduced differentiation into central memory T cells, whereas effector memory T cells were not affected. PONA, however, almost completely blocked differentiation into effector memory cells and reduced the percentage of central memory T cells (Fig. 3A). When looking at the expansion of CD45RO^+^ memory cells upon blinatumomab treatment, significant reduction in absolute cell counts were observed for DASA and PONA (Supplementary Figure S2B).

To further address the immunomodulatory effects of different TKI, cytokine release of PBMCs after blinatumomab treatment with or without concomitant TKI treatment was measured. Supplementary Figure S3 depicts percent changes in cytokine release by TKI treatment of cocultures of PBMC, target cells and blinatumomab. In accordance with the inhibition of T cell activation and proliferation seen by us, DASA, NILO, and PONA inhibited the release of key cytokines such as TNF-alpha, IFN-gamma, and IL-4, IL-6, and IL-10, whereas IMA had no effect or even increased cytokine release (in case of IL-4 and IL-17a). No inhibition was seen for IL-2 and IL-12p70 release. CCL-2 was only affected by DASA at peak doses.

### BCR::ABL1 TKIs inhibit phosphorylation of LCK upon bispecific antibody therapy

TKIs inhibiting BCR::ABL1 activity often interfere with kinases of the Src family (Konig et al. 2008). In order to elucidate the underlying mechanism of T cell inhibition by BCR::ABL1 TKIs phosphoflow experiments were performed. Since the Src kinase lymphocyte-specific protein tyrosine kinase (LCK) plays a major role in T cell activation by phosphorylation of ZAP-70 and itself, we analyzed pY394 LCK phosphorylation in T cells upon blinatumomab stimulation. After 5 min, profound phosphorylation of LCK was observed, which was still detectable after 15 min (Fig. 3B). In the presence of DASA or NILO, LCK phosphorylation was markedly reduced. IMA did not affect LCK phosphorylation. In the presence of PONA, background phosphorylation of LCK was almost completely blocked and no phosphorylation was observed upon blinatumomab stimulation. This underlines the profound inhibitory effect of BCR::ABL1 TKI on blinatumomab-induced T cell activation.

### Reduced tumor cell lysis upon treatment with BCR::ABL1 TKIs

**Fig. 4:**
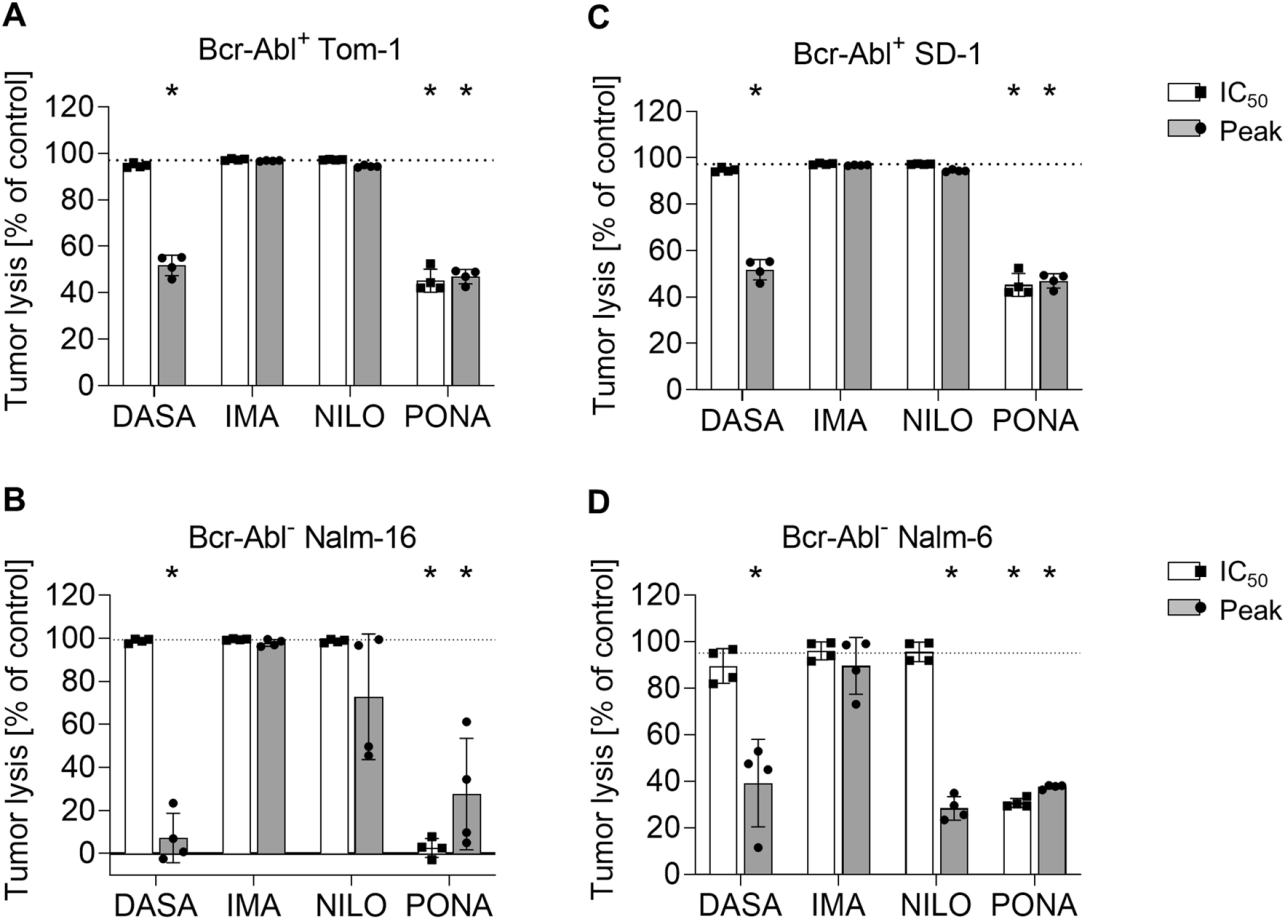
Blinatumomab-induced T cell-mediated target cell lysis in the presence of TKI. Lysis of different ALL cells during 3 day coculture assays with 100,000 PBMC, 100,000 B-ALL cells and blinatumomab at 1 ng/ml was determined by flow cytometry. The following ALL cell lines were utilized: **A** BCR::ABL1^+^ TOM-1, **B** BCR::ABL1^+^ SD-1, **C** BCR::ABL1^−^ Nalm-6, **D** BCR::ABL1^−^ Nalm-16. Statistical analysis with Mann–Whitney *U* test. *p < 0.05

To investigate the effect of TKI on blinatumomab-induced target cell lysis, flow cytometry-based lysis assays were performed. After 3ee days of coculture with PBMCs and blinatumomab, potent lysis of ALL cell lines was observed. Tumor cell lysis was impaired by 50–90% through DASA at peak levels. Likewise, PONA inhibited tumor cell lysis by 50–90% even at IC_50_ doses. NILO only had minor effects on BCR::ABL1^−^ cell lines, but did not inhibit tumor cell lysis of BCR::ABL1^+^ cell lines (Fig. 4). IMA did not exhibit inhibitory properties.

### Tyrosine kinase inhibitors affect blinatumomab efficacy in primary human B-ALL samples

**Fig. 5:**
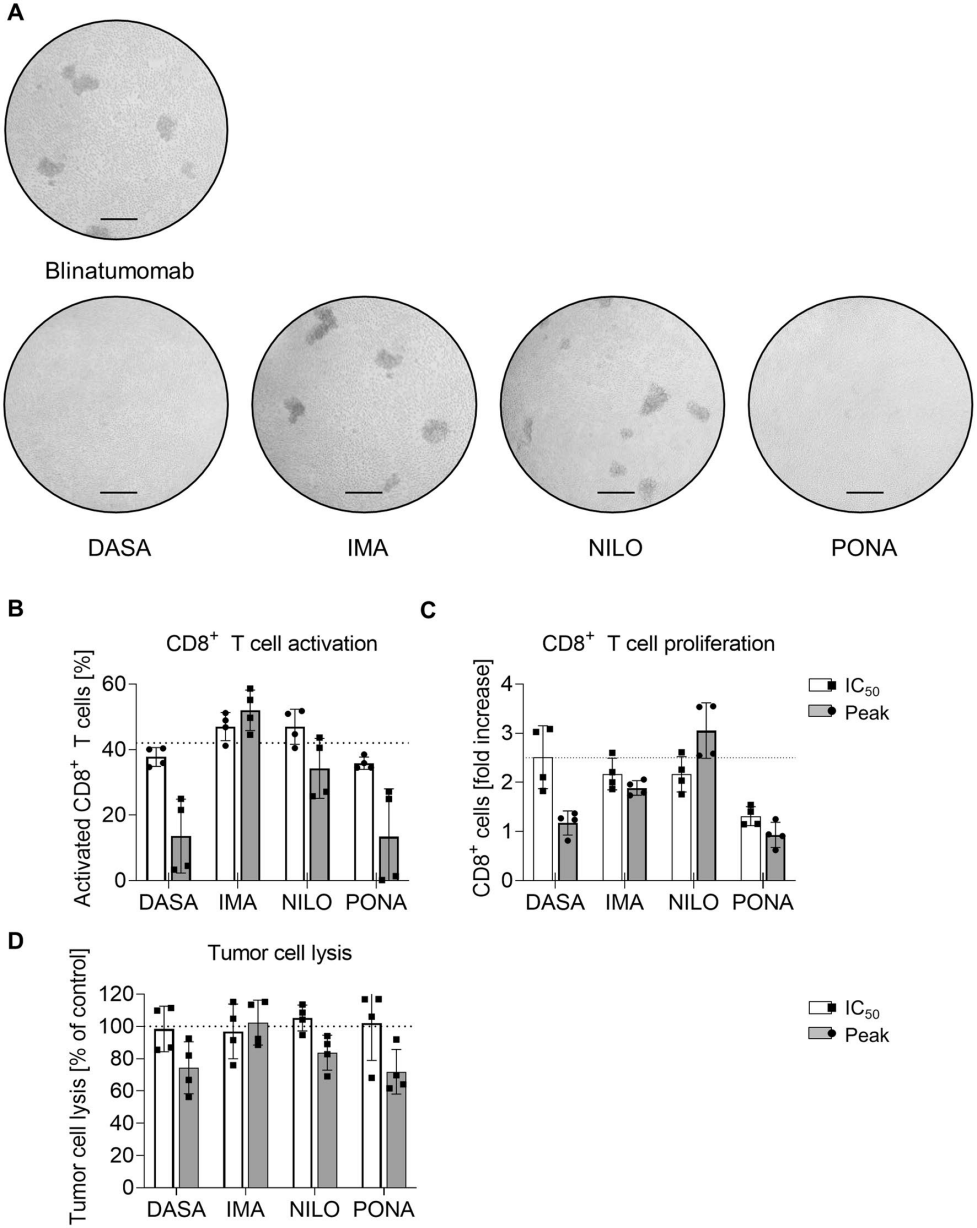
Autologous lysis of B-ALL patient samples in the presence of blinatumomab and TKI. A Exemplary microscopic documentation of T cell cluster formation after 3 day coculture of ALL patient samples treated with blinatumomab and different TKI at peak levels. Scale bar = 10 µm. **B**, **C** Flow cytometric analysis CD8^+^ T cell CD69 expression (**B**) and CD8^+^ T cell proliferation (**C**) after 3 day coculture assays using 200,000 cells/well (*n* = 4) and blinatumomab at 1 ng/ml. **D** Inhibition of lysis of ALL blasts by autologous T cells in the presence of TKI during 3 day coculture assays using 200,000 cells/well (*n* = 4) and blinatumomab at 1 ng/ml. *p < 0.05

To mimic the conditions in the peripheral blood of B-ALL patients more closely, autologous lysis assays were performed using PBMCs from B-ALL patients with > 60% blasts. Patients’ characteristics are shown in Table 1. After three days of coculture with blinatumomab, T cell cluster formation due to activation and expansion were seen. DASA and PONA at peak levels inhibited cluster formation, whereas IMA and NILO did not (Fig. 5A). Again, activation and proliferation of autologous T cells was markedly reduced in the presence of DASA and PONA (Fig. 5B, C). IMA only affected T cell proliferation at peak levels, which remained unaffected by NILO at all. Absolute tumor cell lysis was measured by flow cytometry by gating for CD10^+^CD20^+^ B-ALL blasts (Exemplary gating strategy depicted in Supplementary Figure S1F). Effective lysis was seen in all patients with lysis ranging from 26 to 60%. However, the efficacy of blinatumomab was moderately inhibited by DASA, NILO and PONA at peak levels (Fig. 5D). Synergistic effects, i.e., a more effective lysis by combining TKIs and blinatumomab were not consistently observed (Fig. 5D).

## Discussion

While bsAbs such as blinatumomab have clearly revolutionized the treatment of B-ALL (Topp et al. 2015), the effect of potent BCR::ABL1 inhibition, e.g., by PONA is equally impressive, which in combination with chemotherapy in the frontline setting improved the long-term overall survival up to 80% (Ravandi et al. 2010; Fielding et al. 2014; Chang et al. 2019), compared to 10% in the pre-TKI era (Kantarjian et al. 2000). In contrast to CML, where TKI monotherapy is highly effective, combination regimes are needed to fight B-ALL. Several studies have identified the combination of TKI with blinatumomab as treatment option for BCR::ABL1^+^ B-ALL patients (Assi et al. 2017; Chiaretti et al. 2019; King et al. 2019; Foa et al. 2020); however, uncertainty remains regarding drug sequence, combination and dosing.

Furthermore, TKI may potently affect antitumor immunity by inhibiting signaling pathways necessary for activation of immune effector cells. We here demonstrate that targeted therapy with BCR::ABL1 inhibitors hampers T cell function in vitro to different extent with more pronounced effects with PONA and DASA than with IMA and NILO. Our findings are in line with the data recently provided by Leonard et al. (2021). In addition to the findings by Leonard et al. we further investigated the precise in vitro effects of TKIs on T cell differentiation and cytokine release induced by blinatumomab.

In our hands, DASA and PONA elicited persistent inhibition of blinatumomab-induced T cell proliferation, differentiation, cytokine production and effective killing of B-ALL cells. Inhibition of killing was similar in all four tested cell lines, while activation and proliferation was somewhat preserved in SD-1 cells, possibly pointing to background off-target effects.

The other TKIs affected T cells more heterogeneously. Beyond inhibiting oncogenic BCR::ABL1 signaling, it has already been demonstrated that TKIs like IMA, NILO and DASA impair reactivity of different immune effector cells like dendritic cells, T cells, and natural killer cells (Appel et al. 2005; Blake et al. 2008; Chen et al. 2008; Schade et al. 2008a, b; Fraser et al. 2009). In terms of T cell biology, data is controversial as it has been shown that naive T regulatory cells (Tregs) accumulated after exposure to TKIs pointing to an anti-inflammatory and immunomodulatory effect (Marinelli Busilacchi et al. 2018). Other authors proposed an inhibition of immunosuppressive cells like Tregs, which might improve function of effector T cells (Krusch and Salih 2011). Notably, this may even apply for DASA as highlighted by the findings on clonal lymphocyte expansion in DASA-treated CML patients (Kim et al. 2009; Mustjoki et al. 2009; Kreutzman et al. 2010, 2011; Rohon et al. 2010).

Since inhibitory effects are dose dependent and might only be seen at peak doses as observed with NILO, TKI dosing in combination regimes should be subject to further investigation.

It is known that BCR::ABL1 inhibitors not only affect the BCR::ABL1 fusion protein, but also Src kinases such as C-Src, Lyn and LCK (Schade et al. 2008a, b; Lee et al. 2010). These kinases are of importance for T effector cells (e.g., CD8^+^ cells) (Palacios and Weiss 2004) and we could demonstrate that almost all inhibitors affect phosphorylation and thereby signaling of LCK upon stimulation with blinatumomab.

Regarding side effects like life-threatening cytokine release syndrome (CRS) the combination of blinatumomab with TKIs can be feasible as cytokine production is reduced by some of the TKIs. Of note, IL-2 production was not affected by any of the TKIs, whereas IL-6 release, which is mainly responsible for CRS, was inhibited by DASA, NILO and PONA. In accordance with our data, Mestermann et al. proposed DASA as pharmacological on/off switch for chimeric antigen receptor T (CART) cells, leading to potent reduction of cytokine release (Mestermann et al. 2019).

Our data highlight the potential risks and benefits of the combination of TKIs with blinatumomab and call for more controlled clinical trials. While the combination of DASA/PONA and blinatumomab has been shown to be clinically highly effective (Chiaretti et al. 2019; Couturier et al. 2020; Foa et al. 2020), one might speculate that efficacy could be even more pronounced with IMA or NILO. However, the in vitro off-target effects seem to be less relevant in patients (Sillaber et al. 2009; Rodriguez et al. 2012; Zitvogel et al. 2016). Further in vivo experiments could help to clarify this discrepancy. In summary, correlation with clinical trial data is needed to ensure efficacy of combination regimes comprising bsAbs and TKIs.

## Methods

### Patient samples

All experiments were carried out in accordance with the Helsinki protocol and the Ethics Committee of the University of Tübingen vote (13/2007V) between 2007 and 2020. All experimental protocols were approved by the Ethics Committee of the University of Tübingen (13/2007V). Informed consent was obtained from all patients. Gene nomenclature was carried out in accordance with the HUGO Gene Nomenclature Committee (HGNC) recommendations for the designation of gene fusions (Bruford et al. 2021).

Peripheral blood samples of healthy donors or B-ALL patients were collected and PBMCs were isolated using density gradient centrifugation with Biocoll Cell Separation Solution (Biochrom, Berlin, Germany). The B-ALL cell lines Nalm-6, Nalm-16, SD-1 and TOM-1 were purchased from the German Collection of Microorganisms and Cell Cultures (DMSZ, Braunschweig, Germany) and were routinely tested negative for mycoplasma. PBMCs and cell lines were kept in RPMI 1640 (LifeTechnologies, Darmstadt, Germany) supplemented with 10–20% heat-inactivated fetal calf serum (Biochrom), 1 mmol/l sodium pyruvate (Biochrom), non-essential amino acids (Biochrom), 2 mmol/l l-glutamine (Lonza Group, Basel, Switzerland), 100 U/ml penicillin (Sigma-Aldrich, St.Louis, USA), 100 μg/ml streptomycin (Sigma-Aldrich), and 50 μmol/l beta-mercaptoethanol (Merck, Darmstadt, Germany) at 37 °C and 5% CO_2_.

### Tyrosine kinase inhibitors (TKI)

Dasatinib (DASA), imatinib (IMA), nilotinib (NILO), and ponatinib (PONA) were purchased from Cayman Chemical Company (Ann Arbor, USA). After dissolving in DMSO, TKI were kept at – 20 °C until use. Stock concentrations were 20 mM for DASA, IMA and PONA, and 3 mM for NILO, respectively. Peak and IC_50_ concentrations were chosen according to the literature: DASA 150 nM (peak) and 10 nM (IC_50_); IMA 3000 nM (peak) and 600 nM (IC_50_); NILO 3000 nM (peak) and 30 nM (IC_50_); PONA 145 nM (peak) and 70 nM (IC_50_) (Peng et al. 2004; Kantarjian et al. 2006, 2010; Rix et al. 2007; Weisberg et al. 2007; Cortes et al. 2012; Menna et al. 2020). Direct effects of DMSO (maximum level was 0.1% v/v in NILO peak samples) were ruled out by including DMSO only as control throughout all experiments.

### Thymidine incorporation assays

For determination of T cell proliferation, ^3^H thymidine incorporation assays were performed. 100,000 PBMCs, 100,000 irradiated target cells, blinatumomab at 1 ng/ml and TKI were incubated for 2 days in 96-well plates followed by 20 h incubation in the presence of ^3^H-methyl thymidine (0.5 μCi/well). After harvesting on filter mats, incorporated ^3^H-methyl thymidine was measured using a liquid scintillation counter (MicroBeta2 2450 Microplate counter, PerkinElmer, Waltham, USA).

### Flow cytometry

CD4-PacificBlue (clone OKT4; 1:100), CD8-FITC (clone RPA-T8; 1:100), CD10-APC/Cy7 (clone HI10a, 1:100), CD69-PE (clone FN50; 1:200), CD276-PE/Cy7 (clone MIH42; 1:100) and the respective isotype control antibodies were purchased from BioLegend (San Diego, USA). CD45-AmCyan (clone 2D1, 1:200) was purchased from BD Biosciences (Franklin Lakes, USA).

For flow cytometry-based lysis assays, 100,000 target cells were incubated in 96 well plates together with 100,000 PBMCs, blinatumomab at 1 ng/ml and different tyrosine kinase inhibitors at the indicated concentrations. After 3 days, flow cytometric analysis was performed. Nalm-6 cells were defined as CD45^−^CD10^+^, Nalm-16 as CD45^−^CD10^+^, SD-1 per cell size and as CD22^+^, TOM-1 as CD45^−^CD10^+^, T cells as CD45^+^CD4^+^ or CD45^+^CD8^+^, and activated subsets were identified using CD69 as surrogate marker. Absolute cell numbers were determined by the acquisition of equal amounts of compensation particles (BD Biosciences) per sample, thus allowing for calculation of the absolute degree of tumor cell lysis. Binding of antibodies to Fc receptors was blocked with Flebogamma DIF (Grifols, Barcelona, Spain) at 50 μg/ml. Data acquisition was performed using a FACSCanto II (BD Biosciences). For data analysis, FlowJo_V10 software (Tree Star, Ashland, OR) was utilized.

### Legendplex cytokine arrays

Legendplex cytokine arrays (Human Essential Immune Response, Biolegend) were performed according to the manufacturer’s protocol by utilizing supernatants from lysis assays as described above.

### Phosphoflow

For phosphoflow experiments, T cells were isolated by magnetic-activated cell sorting (MACS) using the Pan T cell isolation kit according to the manufacturer’s protocol (Miltenyi Biotech, Köln, Germany). T cells were then cultured in RPMI 1640 media and different TKI at peak concentrations overnight. 96-well flat bottom high protein binding plates (Nunc Maxisorp, ThermoFisher) were coated overnight with blinatumomab at 100 ng/ml in PBS. T cells were transferred to FCS with different TKI present at peak concentrations and stimulated on 96-well plates. After 0, 5, and 15 min, signalling was stopped by buffer 1 of the Perfix Expose Kit (Beckman Coulter) and T cells were further treated according to the manufacturer’s protocol. Intracellular staining was performed with an anti-LCK(pTyr394) antibody (Genetex, 1:500) and anti-rabbit-PE secondary antibody (Cell Signaling Technologies, 1:200). Data acquisition was performed using a FACSCanto II (BD Biosciences).

### Statistical analysis

Data are displayed as bar diagram with mean values overlaid by dot plots. Mann–Whitney U tests were used to compare individual groups. Statistical analyses were conducted using GraphPad Prism 8.1.0 software. *P* values of < 0.05 were considered statistically significant.

**Table 1:** Patients’ characteristics

Patient	Age	ALL type	Karyotype	FISH t(9;22)	Break point	Risk group	PBB % Diff	WBC [G/l]	Hb [g/dl]	Plt [G/l]
#1	22	Pre-B	46,XY[30]	Neg	–	HR	95	74.14	13,2	13
#2	66	C	n.d.	Neg	–	HR	88	300.95	13.3	121
#3	21	C	n.d.	Pos	m-BCR	VHR	87	463.01	11.6	31
#4	68	C	45,XY,-7,t(9;22)(q34;q11.2),del(11)(p10),+2~3mar,inc[6]/46,XY[19]	Pos	M-BCR	VHR	79	29.00	13.1	28
#5	76	C	47,XY,t(2;16)(p11;p11),+der(8)t(8;8)(p23;q23), der(8)t(8;8)(p23;q23),t(9;22)(q34;q11)[8]; 47,XY,t(2;16)(p11;p11),+der(8)t(8;8)(p23;q23), der(8)t(8;8)(p23;q23),t(9;22)(q34;q11)[8]; 47,XY,t(2;16)(p11;p11),+der(8)t(8;8)(p23;q23), der(8)t(8;8)(p23;q23),t(9;22)(q34;q11)[8]; 48,XY,+X,t(2;16)(p11;p11)der(8)t(8;8)(p23;q23), t(9;22)(q34;q11)+der(22)t(9;22)(q34;q11)[2]; 46,XY[9]	Pos	m-BCR	VHR	82	68.75	9.6	20

ALL, acute lymphoblastic leukemia; C, common; n.d., not determined; Neg., negative; Pos., positive; m-BCR., minor breakpoint cluster region; M-BCR., major breakpoint cluster region; FISH, fluorescence in situ hybridization; HR, high risk; VHR, very high risk; PBB, peripheral blood leukemic blasts; Diff, differential blood count; WBC, white blood count; Hb, hemoglobin; Plt, platelets

## Supplementary Information

Below is the link to the electronic supplementary material.

Supplementary file1 (PDF 358 kb)

## Declarations

### Additional information

Data sharing upon reasonable request to the corresponding author.
