# Supplementary material for: BCR::ABL1 tyrosine kinase inhibitors hamper the therapeutic efficacy of blinatumomab in vitro
Source: J Cancer Res Clin Oncol. 2022 May 13;148(10):2759–71. doi: 10.1007/s00432-022-04039-5 (PMC9470724; doi:10.1007/s00432-022-04039-5)
Supplement: Supplementary file 1 — Supplementary file1 (PDF 358 kb) [file 432_2022_4039_MOESM1_ESM.pdf]

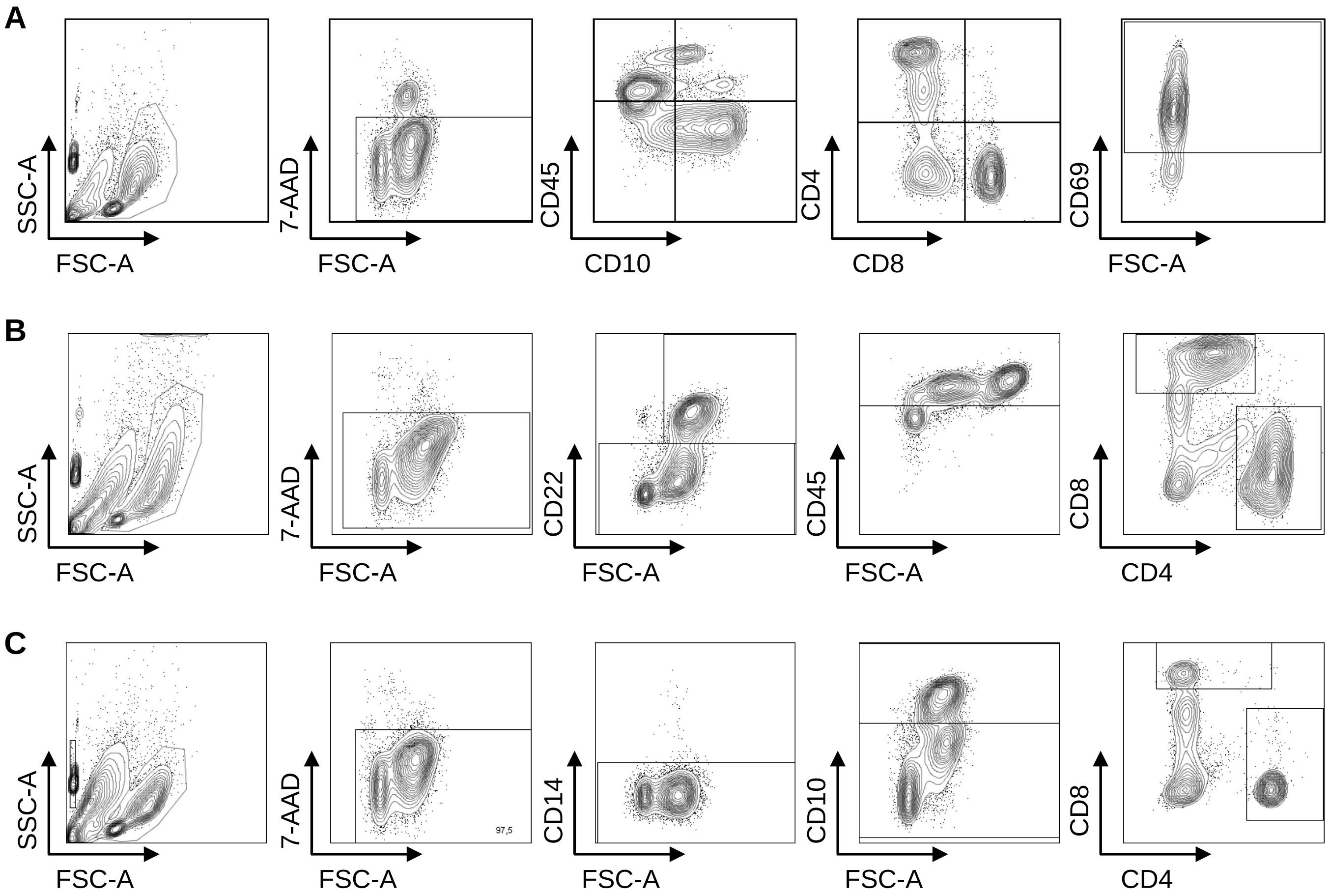

Figure S1: Exemplary gating strategies for flow cytometry based assays

Exemplary gating strategy for 3 day coculture assays using 100,000 PBMC, 100,000 ALL cells and blinatumomab at 1 ng/ml: **A** Tom-1: Cells, viable (7-AAD-), CD45-CD10+ TOM-1; CD45+CD4+ or CD45+CD8+ T cells, CD69+ T cells. **B** SD-1: Cells, viable (7-AAD-), CD22+ SD-1; CD45+CD4+ or CD45+CD8+ T cells, CD69+ T cells. **C** Nalm-6: Cells, viable (7-AAD-), CD14-CD10+ Nalm-6; CD45+CD4+ or CD45+CD8+ T cells, CD69+ T cells.

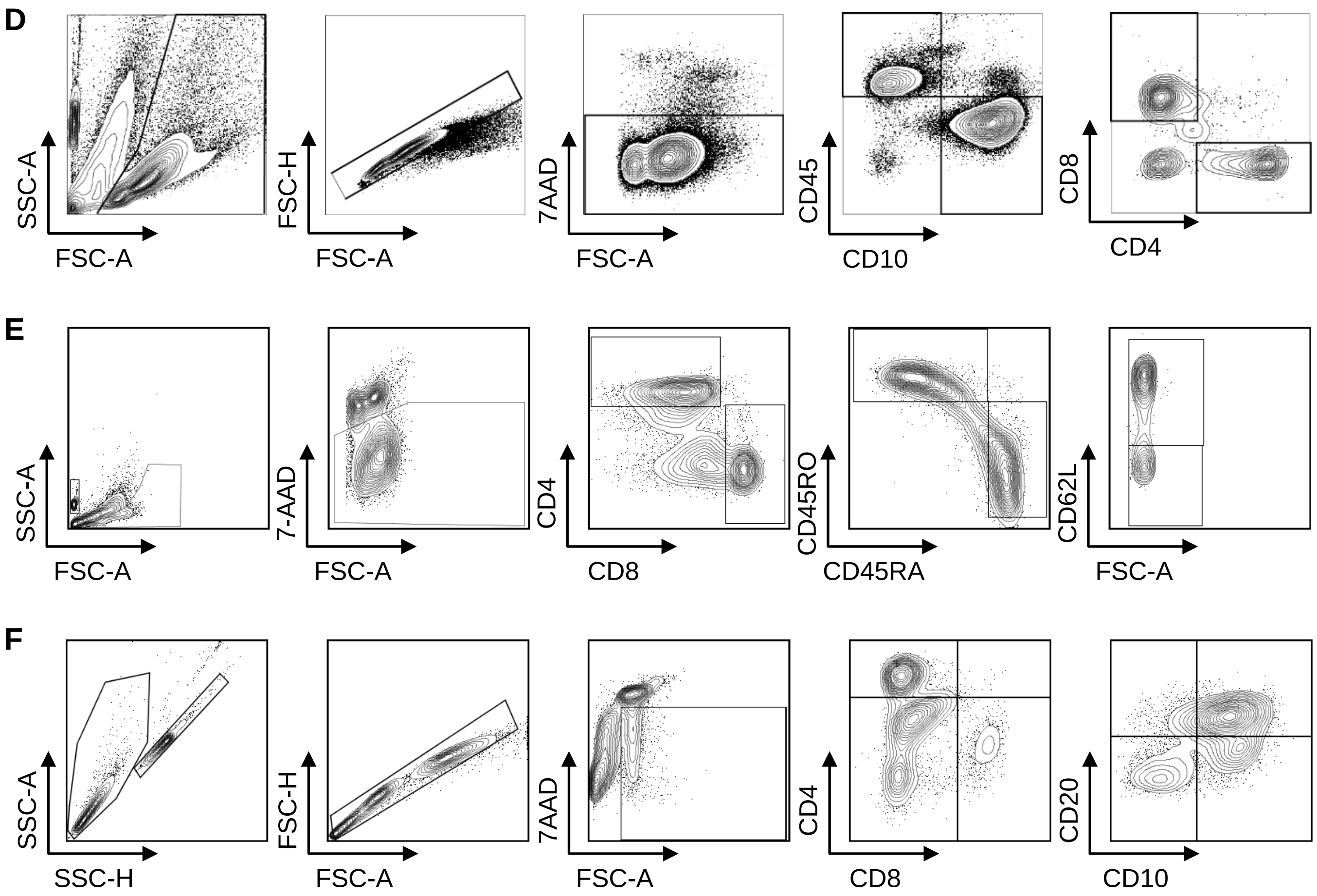

Figure S1 (continued): Exemplary gating strategies for flow cytometry based assays

Exemplary gating strategy for 3 day coculture assays using 100,000 PBMC, 100,000 ALL cells and blinatumomab at 1 ng/ml: **D** Nalm-16: Cells, viable (7-AAD-), CD45-CD10+ Nalm-16; CD45+CD4+ or CD45+CD8+ T cells, CD69+ T cells. **E** Flow cytometric analysis of T cell differentiation: cells, viable (7-AAD-), CD4+ or CD8+, naive (CD45RA+CD62L+), effector (CD45RA+CD62L-), central memory (CD45RO+CD62L+) or effector memory T cells (CD45RO+CD62L-). **F** Flow cytometry based autologous lysis assays using ALL patient samples: cells, singlets, viable (7-AAD-), CD4+ or CD8+, CD20+CD10+ B-ALL blasts

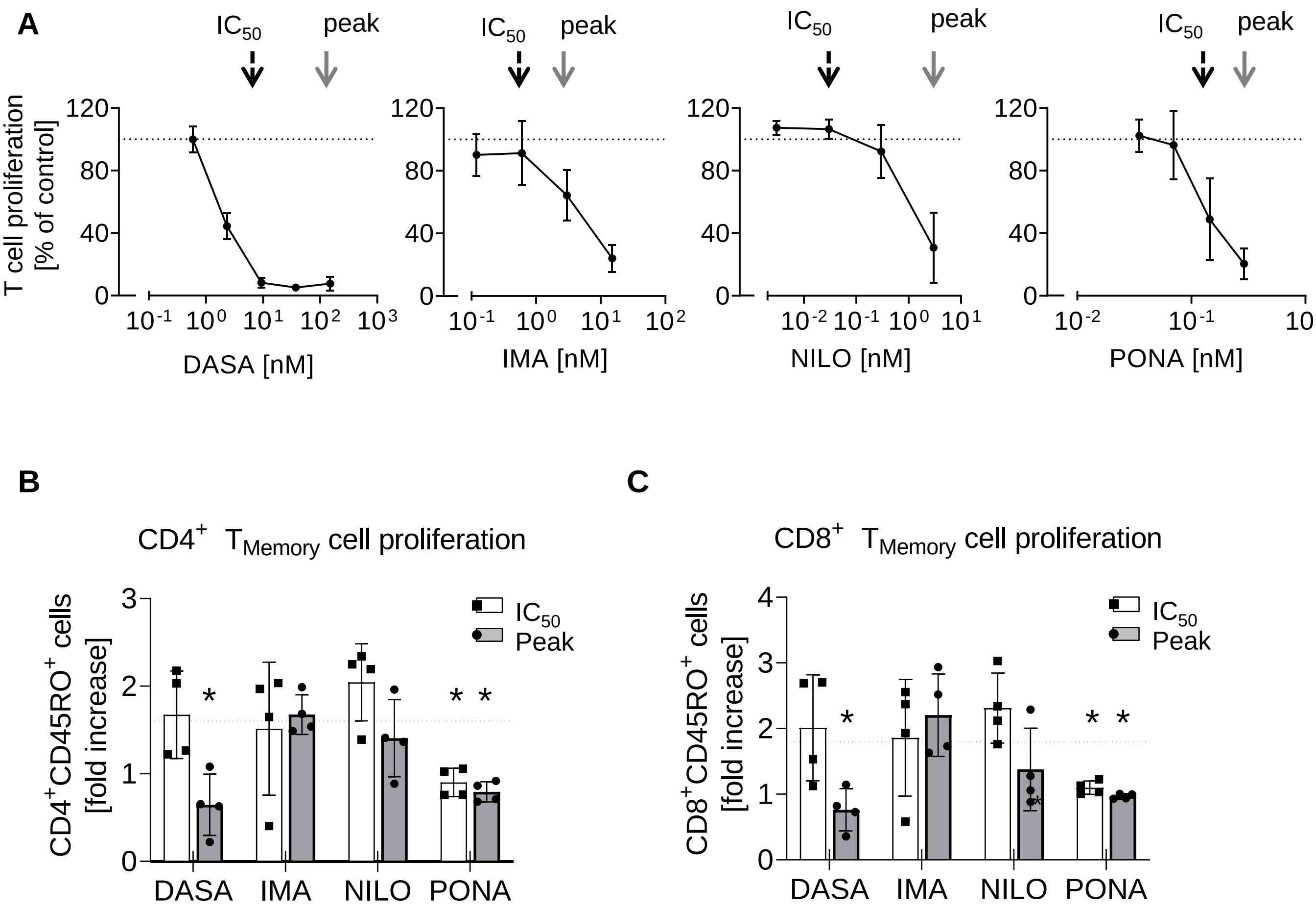

Figure S2: Impact of BCR::ABL1 TKI on T cell proliferation and differentiation induced by blinatumomab and B-ALL cells.

A 3 day ^{3}H Thymidine incorporation assays (n=3) using 100,000 y-irradiated PBMC, 100,000 BCR::ABL1$^+$ TOM-1, blinatumomab at 1 ng/ml and TKI at different concentrations were performed. Arrows depict IC_{50} and plasma peak levels. **B-C** Expansion of CD45RO Memory subsets after during 3 day coculture assays using 200,000 B-ALL patient-derived peripheral blood mononuclear cells/well (n=4) and blinatumomab at 1ng/ml. Statistical analysis with Mann-Whitney U test.

A

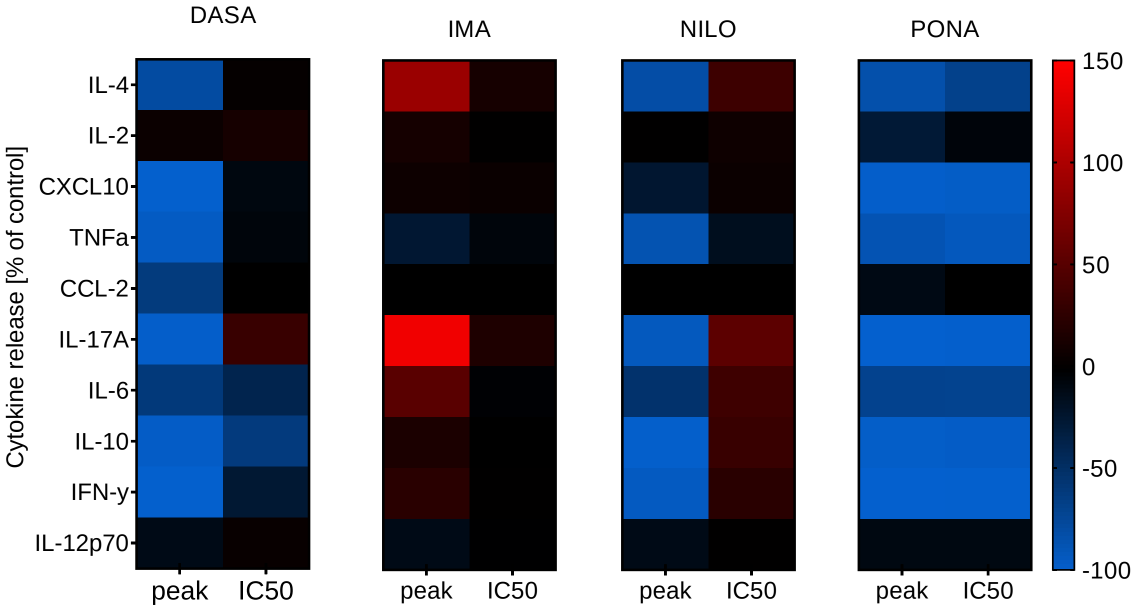

Figure S3: Impact of TKI on blinatumomab-induced cytokine release of T cells.

A Legendplex cytokine arrays were performed using supernatants from 3 day coculture assays with 100,000 PBMC, 100,000 BCR::ABL1^{+} TOM-1 and blinatumomab at 1ng/ml. Heatmap colors reflect % changes in cytokine release relative to cytokine release with PBMC, target cells and blinatumomab (set to 100%).
